# Crosstalk between Cancer Cells and Cancer-Associated Fibroblasts Mediated by TGF-β1–IGFBP7 Signaling Promotes the Progression of Infiltrative Gastric Cancer

**DOI:** 10.3390/cancers15153965

**Published:** 2023-08-04

**Authors:** Zhijun Hong, Wen Xie, Huiqin Zhuo, Xujin Wei, Kang Wang, Jia Cheng, Lingyun Lin, Jingjing Hou, Xin Chen, Jianchun Cai

**Affiliations:** 1 Department of Gastrointestinal Surgery, Zhongshan Hospital of Xiamen University, School of Medicine, Xiamen University, Xiamen 361004, China; hongzhijun@stu.xmu.edu.cn (Z.H.); xiewen@stu.xmu.edu.cn (W.X.); zhuohuiqin@xmu.edu.cn (H.Z.); wkang0910@stu.xmu.edu.cn (K.W.); drchengjia@163.com (J.C.); lingyunlin@xmu.edu.cn (L.L.); jjhou@xmu.edu.cn (J.H.); 2 Xiamen Municipal Key Laboratory of Gastrointestinal Oncology, Xiamen 361004, China; 3 Institute of Gastrointestinal Oncology, Medical College of Xiamen University, No. 201-209, Hubin South Road, Xiamen 361004, China; weixujin@fjmu.edu.cn (X.W.); drklaus@fjmu.edu.cn (X.C.); 4 The Graduate School, Fujian Medical University, Fuzhou 350004, China

**Keywords:** gastric cancer, metastasis, Ming classification, cancer-associated fibroblast, IGFBP7, TGF-β1

## Abstract

**Simple Summary:**

This study clarified that in the tumor microenvironment, tumor cell-derived TGF-β1 induces the appearance of an IGFBP7^+^ CAFs subgroup, and its higher IGFBP7 extracellular secretion level accelerated the progression of tumors. Our work suggests that IGFBP7 is a novel predictive marker for clinical outcomes in gastric cancer patients.

**Abstract:**

Patients with infiltrative-type gastric cancer (GC) (Ming’s classification) have a poor prognosis due to more metastasis and recurrence. Cancer-associated fibroblasts (CAFs) in infiltrative-type extracellular matrix (ECM) have specific characteristics compared with those of expansive types with respect to metastasis, but the mechanism is still unclear. Based on our proteomics data, TCGA data analysis, and immunohistochemical staining results, significantly higher expression of IGFBP7 was observed in GC, especially in the infiltrative type, and was associated with a poor prognosis. Combining single-cell transcriptome data from GEO and multiple immunofluorescence staining on tissue showed that the differential expression of IGFBP7 mainly originated from myofibroblastic CAFs, the subgroup with higher expression of PDGFRB and α-SMA. After treating primary normal fibroblasts (NFs) with conditional medium or recombined protein, it was demonstrated that XGC-1-derived TGF-β1 upregulated the expression of IGFBP7 in the cells and its secretion via the *P*-Smad2/3 pathway and mediated its activation with higher FAP, PDGFRB, and α-SMA expression. Then, either conditional medium from CAFs with IGFBP7 overexpression or recombined IGFBP7 protein promoted the migration, invasion, colony formation, and sphere growth ability of XGC-1 and MGC-803, respectively. Moreover, IGFBP7 induced EMT in XGC-1. Therefore, our study clarified that in the tumor microenvironment, tumor-cell-derived TGF-β1 induces the appearance of the IGFBP7^+^ CAF subgroup, and its higher IGFBP7 extracellular secretion level accelerates the progression of tumors.

## 1. Introduction

Gastric cancer (GC) is a significant global health issue, with over 1 million new cases and 769,000 deaths in 2020. The highest incidence rates are seen in Eastern Asia [1]. The tumor microenvironment (TME) is composed of various components, including immune, inflammatory, endothelial, adipose, and fibroblast cells. These components release signaling molecules, such as growth factors and cytokines, that influence the maintenance and acquisition of characteristics of cancer stem cells (CSCs) within the TME. These complex interactions promote gastric cancer progression, leading to invasive and metastatic phenotypes [2,3,4,5].

Cancer-associated fibroblasts (CAFs) can arise from multiple sources, including normal fibroblasts (NFs), epithelial cells, endothelial cells, adipocytes, pericytes, cancer stem cells, hematopoietic stem cells, and bone marrow stromal cells [6]. The diverse origin of CAFs accounts for their heterogeneous features in gastric cancer [7,8,9,10]. Currently, there are several commonly used markers for CAF, such as platelet-derived growth factor receptor alpha (PDGFRα), platelet-derived growth factor receptor beta (PDGFRβ), alpha-smooth muscle actin (α-SMA), and fibroblast activating protein (FAP) [11]. However, these markers are usually only expressed in a subset of fibroblasts within the tumor and not in all CAFs. Moreover, the CAF subtypes involved in cancer progression may vary depending on the cancer type, as they have different carcinogenic and progression mechanisms.

The name “insulin-like growth factor binding protein-7 (IGFBP7)” was given due to the similarity of the amino acid sequence of the precursor peptide to the IGFBP family as a whole and its lower affinity to insulin-like growth factor (IGF) when compared specifically to IGFBP3 [12]. IGFBP7 is notably elevated in undifferentiated tumors, indicating its potential role in tumor differentiation [13]. The median level of IGFBP7 mRNA is also significantly increased in GC compared to paired normal tissues. Additionally, IGFBP7 expression is positively correlated with tumor invasion depth, lymph node metastasis, distant metastasis/recurrence, and pathological staging in GC patients [14]. Collectively, IGFBP7 appears to be a valuable prognostic marker in GC, despite the lack of clarity regarding its underlying mechanisms.

In this study, we analyzed the expression pattern of IGFBP7 using data from TCGA and GEO databases and conducted immunohistochemistry experiments to investigate its correlation with clinical prognosis. Our results revealed that IGFBP7 is primarily expressed in the tumor stroma. Subsequently, we explored the interaction mechanism between IGFBP7 and primary fibroblasts in GC cells and conducted functional experiments to examine the role of secreted IGFBP7 in GC cells.

## 2. Materials and Methods

### 2.1. Quantitative Proteomics Analysis

Fresh GC tissue and adjacent tissue were both treated with a lysis buffer. Peptides were concentrated and washed on a C18 column (Thermo Scientific, Waltham, MA, USA). The peptides were then subjected to vacuum centrifugation for drying. The resulting tryptic peptides were reconstituted in 0.1% formic acid. The dissolved peptides were analyzed using an LTQ-Orbitrap XL instrument (Thermo Scientific). The full scan range for m/z was set from 370 to 1700.

For the identified differentially expressed proteins, the significantly upregulated 45 for GO/KEGG enrichment analysis using the R package “clusterProfiler” was selected.

### 2.2. Retrieval and Analysis of TCGA Transcriptome Sequencing Data

Data from RNA sequencing of 33 types of tumors were downloaded from The Cancer Genome Atlas (TCGA) database (https://portal.gdc.cancer.gov) on 20 March 2022, and the data were extracted in TPM format. Clinical data and gene expression profiles for TCGA-STAD bulk RNA sequencing dataset were also downloaded. The gene expression matrix was normalized using log2 (TPM + 1 ), and differential expression analysis was performed using the DESeq2 (v1.30.1) package to compare the gene expression levels between GC and normal mucosal epithelial tissue. The Spearman rank correlation test was used to perform gene expression correlation analysis. Patients were divided into high- and low-expression groups based on selected gene expression levels, and the Kaplan–Meier survival curve was used to estimate the prognosis of the two groups, including their median survival time. This study used the “estimate” R package version 1.0.13 to calculate the immune infiltration score for the TCGA-STAD data. To calculate the immunodeficiency status, we used the ssGSEA algorithm provided in the GSVA R package version 1.46.0.

### 2.3. Collection and Analysis of Single-Cell RNA Sequencing Data

We downloaded the scRNA-seq dataset (GSE183904) of GC patients from the Gene Expression Omnibus (GEO) database and selected six diffuse GC (DGC) and ten normal tissue sequencing data from it. These 16 matrices were used as input objects for Seurat (v4.0.1), and “Scrublet (v0.2.3)” was used to remove doublets. Then, the UMI count data matrix was processed using the “SCTransform” workflow. “Harmony (v0.1.0)” was used to correct batch effects between samples. The “RunPCA” function was used for unsupervised principal component analysis, and the first 20 principal components were used for “FindNeighbors” to perform unsupervised clustering of cells. The “RunUMAP” function was used to perform UMAP visualization of the clustering results of cells. Finally, this study used the “FindClusters” function to identify cell clusters with a resolution of 0.6. To determine differentially expressed genes (DEGs), we used default parameters to analyze DEGs using the “FindMarkers/FindAllMarkers” functions and the Wilcoxon rank-sum test method. Cells were then identified and annotated based on cell marker genes and differentially expressed genes.

### 2.4. Clinical Samples and Tissue Microarray

In this study, we collected 10 GC samples from the Zhongshan Hospital of Xiamen University for research purposes and obtained informed consent and signed relevant documents from all patients. The process was approved by the Medical Ethics Committee affiliated with Zhongshan Hospital of Xiamen University. The tissue microarray was of GC samples (HStmA160CS01, OUTDO Biotech, Shanghai, China) and came with clinical information.

### 2.5. Cell Culture and Treatment

The human GC cell lines MGC-803 (Chinese Academy of Science, Shanghai, China) and XGC-1 (Infiltrating GC cell line constructed by our research group [15], China patent number: CN103396994A) were cultured in Roswell Park Memorial Institute (RPMI)-1640 (Biological Industries, Beit Haemek, Israel) supplemented with 10% fetal bovine serum (Sigma, St. Louis, MO, USA) and 100 U/mL penicillin and 100 μg/mL streptomycin (Biological Industries, Kibbutz Beit Haemek, Israel) at 37 °C with 5% CO_2_. Primary fibroblast cells were cultured in Dulbecco’s Modified Eagle Medium/Nutrient Mixture F-12 (DMEM/F12) (Biological Industries) basal medium. The purchased cells all had Short Tandem Repeats validation reports and were free from mycoplasma contamination.

The surgical specimens of GC and normal gastric mucosa were cut into small pieces, digested with 0.1% type II collagenase, and filtered through a 100 μm cell strainer (Corning, Lowell, MA, USA) before being seeded in culture dishes to extract primary fibroblasts. The primary fibroblasts were first identified with morphological examination and then tested for expression of vimentin, fibroblast activation protein (FAP), and alpha-smooth muscle actin (α-SMA) with immunofluorescence (IF). CAFs expressed more FAP and α-SMA than NFs, while both expressed vimentin.

The full-length human IGFBP7 gene sequence was inserted into the pLVX-Puro vector, which was then packaged into a lentivirus with psPAX2 and pMD2.G. The empty pLVX-Puro vector was used as a control. Infection of CAFs with pLVX-Puro-IGFBP7 lentivirus resulted in overexpression of IGFBP7 protein (CAF-IGFBP7), which was confirmed through puromycin selection and Western blot experiments. The pLKO.1 lentiviral vector was used to introduce short hairpin RNA (shRNA) targeting the IGFBP7 gene or control sequence (CCTAAGGTTAAGTCGCCCTCG). For the IGFBP7 gene, two target sequences were employed: IGFBP7 shRNA1 (GCTGGTATCTCCTCTAAGTAA) and IGFBP7 shRNA2 (GTCACTATGGAGTTCAAAGGA).

### 2.6. Conditioned Medium

When XGC-1, CAF, and CAF-IGFBP7 cells reached 80% confluency in a 6 cm dish, serum-free medium was used to culture for an additional 36 h. Afterward, the supernatant was collected. The collected conditioned medium (CM) was centrifuged at 1000 rpm for 5 min at 4 °C and filtered through a 0.45 μm filter. Then, it was concentrated by a 3 kDa molecular weight cutoff filter at 4000 rpm. The concentrated CM was used for subsequent experiments.

### 2.7. Transwell Migration and Invasion Assay

XGC-1 and MGC-803 cells were cultured in conditioned media (CM) collected from CAF and CAF-IGFBP7, rh-IGFBP7, and control. The required Transwell chambers (Corning) for the experiment had a pore size of 8 μm. For the invasion assay, the chambers were coated with a mixture of matrigel and pre-cooled serum-free medium at a 1:3 ratio and placed in a 37 °C cell culture incubator for 1 h to polymerize the mixture into a gel. The cell density was adjusted to 5 × 105 cells/mL. A total of 600 µL of medium containing 10% FBS was added to the lower chamber of a 24-well plate. Then, 200 µL of the cell suspension was added to the Transwell chamber for the migration assay. Cells were cultured for 16 h. For the invasion assay, cells were cultured for 24 h. The cells inside the chamber were wiped with a cotton swab. The external cells were fixed with 4% paraformaldehyde for 10 min and then stained with 0.1% crystal violet for 15 min.

### 2.8. Colony Formation Assays and Tumorsphere Culture

Digestion of XGC-1 and MGC-803 was performed as described in Section 2.6. First, 1000 cells were seeded in the wells of a 6-well plate and cultured for 10 days with media changed every 3 days. Cells were fixed with 4% paraformaldehyde and stained with 0.1% crystal violet. For the tumorsphere culture, sphere media was prepared by adding 2% B27, 20 ng/mL EGF, 10 ng/mL bFGF, 100 IU/mL penicillin, and 100 μg/mL streptomycin to DMEM/F12 medium. XGC-1 and MGC-803 were seeded in ultra-low-attachment 96-well plates (Corning) at a density of one cell per well. A total of 50 μL sphere media was added every 3 days, and sphere growth was observed for 10 days. The number and relative size of the spheres in each group were recorded. Relative sphere volumes were calculated at the same time points using the following equation: volume = length × (width)^2^ × π/6. 

### 2.9. Immunofluorescence

The cells were seeded into 8-well chamber slides and cultured to reach 60–80% confluency. The cells were fixed with 4% paraformaldehyde at room temperature (RT) for 15 min and permeabilized with 0.3% Triton X-100 at RT for 5 min. The cells were blocked with 3% BSA solution at RT for 1 h, followed by overnight incubation with vimentin (ab92547, Abcam, Cambridge, MA, USA), PDGFRB (3169, CST, Danvers, MA, USA), FAP (66562, CST), α-SMA (19245, CST), or IGFBP7 (ab171085, Abcam) primary antibody at 4 °C. The corresponding Alexa Fluor 488 (ab150077, Abcam) or 594 (ab150080, Abcam) conjugated secondary antibody was incubated at RT in the dark for 1 h, and 4′,6-diamidino-2-phenylindole(DAPI)-containing mounting medium (MA0236, Meilunbio, Dalian, China) was used to mount the slides. Images were captured using confocal microscopy (Zeiss LSM780).

### 2.10. Immunohistochemistry (IHC)

After processing tissue chips and formalin-fixed paraffin-embedded (FFPE) samples through a series of deparaffinization steps, including baking, xylene, and gradient alcohol, antigen retrieval was performed using citrate buffer. A 3% hydrogen peroxide solution was applied for 10 min, followed by blocking with goat serum for 10 min. The IGFBP7 primary antibody was incubated at 4 °C overnight. Subsequent staining was performed with an immunohistochemistry kit (Maixin Biotech, Fuzhou, China).

The staining area was divided into four grades based on the percentage of stained area over total tissue: 1 = <25%, 2 = 25–50%, 3 = 50–75%, and 4 = ≥75%. Staining intensity was divided into four grades: 0 (no staining), 1 (weak positivity), 2 (moderate positivity), and 3 (strong positivity). The combination scores were multiplied, producing a weighted score (ranging from 0 to 12). To conduct statistical analysis, a combination score > 6 was defined as high expression, while a score ≤ 6 was considered low expression. Two independent pathologists evaluated the results based on staining intensity and degree. 

### 2.11. *RNA Extraction and Quantitative PCR Analysis*

Total RNA was isolated from cells using TRIzol (Invitrogen) following the manufacturer’s instructions. The extracted RNA was then reverse-transcribed into cDNA using the SuperScript First-Strand Synthesis System for RT-PCR (Invitrogen). For qPCR analysis, Platinum SYBR Green qPCR SuperMix (Invitrogen) was employed. The housekeeping gene GAPDH was used as the internal control. The following primer sequences for quantitative PCR were used: 

IL11: 5′-GAACTGTGTTTGCCGCCTG-3′ (forward),

5′-AGCACACCTGGGAGCTGTA-3′ (reverse);

IL6: 5′-TCCTTCTCCACAAGCGCC-3′ (forward),

5′-GGGCGGCTACATCTTTGGAA-3′ (reverse);

LIF: 5′-TCTTGGCGGCAGTACACAG-3′ (forward),

5′-CGACTATGCGGTACAGCTCC-3′ (reverse);

CXCL14: 5′-ATGAAGCCAAAGTACCCGCA-3′ (forward),

5′-CTTCGTAGACCCTGCGCTT-3′ (reverse);

GAPDH: 5′-TCTCCTCTGACTTCAACAGCGA-3′ (forward),

5′-GTCCACCACCCTGTTGCTGT-3′ (reverse).

### 2.12. Western Blot

The collected cells were lysed using RIPA buffer supplemented with protease inhibitors. The lysate was centrifuged at 12,000 rpm for 15 min. Protein was extracted for 10% SDS-PAGE followed by transfer onto a PVDF membrane. The membranes were blocked with skim milk in TBST (5%, w/v) for 1 h and incubated with the primary antibody overnight. The membranes were incubated with an HRP-conjugated secondary antibody at RT for 1 h and visualized using ECL substrate (1705061, Bio-Rad Laboratories Inc., Hercules, CA, USA).

### 2.13. Enzyme-Linked Immunosorbent Assay (ELISA)

Cells were cultured in a 6 cm dish for 24 h. The medium was replaced with 3 mL serum-free medium and continued to culture for another 24 h. The supernatant was collected and processed according to the protocol of the IGFBP7 ELISA kit (R&D Systems, Minneapolis, MN, USA). The protein level of IGFBP7 in the sample was analyzed by measuring the absorbance at 450 nm using a microplate reader.

### 2.14. Statistical Analysis

The chi-square test was used to analyze the correlation between IGFBP7 expression levels and clinical pathological variables. The Wilcoxon rank-sum test was used to compare the median expression levels of IGFBP7 mRNA with various clinical pathological variables. Kaplan–Meier curves were plotted to assess the impact of IGFBP7 expression on the survival of GC patients. The log-rank test was used to assess the prognostic significance of IGFBP7 and other survival-related factors. A *p*-value less than 0.05 was considered statistically significant in all tests. The statistical analysis was performed using R software (v4.1.2).

## 3. Results

### 3.1. IGFBP7 Is Significantly Highly Expressed in GC, and Its Expression Is Closely Associated with GC Progression

**Figure 1:**
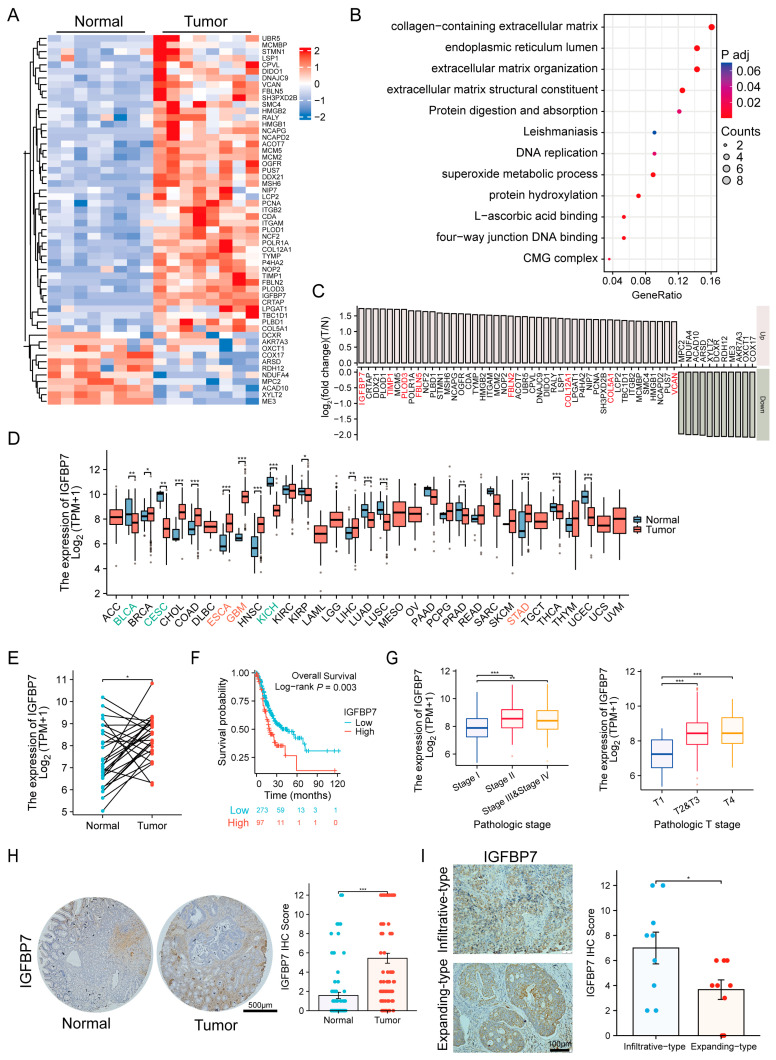
IGFBP7 is highly expressed in gastric cancer (GC) tissue, and its expression is closely related to the occurrence and development of disease. (**A**) A heatmap is presented to display the differentially expressed proteins in the proteomic data of 8 pairs of gastric cancer and matched normal tissues. (**B**) Gene Ontology (GO) and Kyoto Encyclopedia of Genes and Genomes (KEGG) analyses were performed for 45 upregulated proteins in gastric cancer. (**C**) The 45 upregulated proteins and 11 downregulated proteins were sorted based on their fold change values. The most significant collagen-containing extracellular matrix in the GO analysis is highlighted in red. (**D**) Pan-cancer analysis of IGFBP7 mRNA expression using The Cancer Genome Atlas (TCGA) data. (**E**) Paired-sample analysis of IGFBP7 mRNA high expression in GC using TCGA-STAD (GC) dataset. (**F**) TCGA-STAD analysis shows that high expression of IGFBP7 mRNA is associated with poor clinical outcomes in terms of OS, as well as (**G**) advanced clinical pathological staging. (**H**) Immunohistochemistry (IHC) staining shows higher IGFBP7 protein expression in GC samples compared to adjacent non-cancerous tissue. (**I**) IHC staining reveals more IGFBP7 expression in infiltrative type GC compared to expansive type GC. *: *p* < 0.05, **: *p* < 0.01, ***: *p* < 0.001, ns: not significant.

Eight GC tumor samples and paired adjacent normal tissues were collected for TMT-based proteomic analysis to screen for differences in human GC. A total of 624 proteins were identified using log_2_ (T/N fold change) ≥1 or ≤−1, with *p* < 0.05. A total of 45 proteins with the most upregulation and 11 proteins with the most downregulation were obtained (Figure 1A). Subsequently, we selected 45 upregulated proteins to perform GO and KEGG analysis and found that the collagen-containing extracellular matrix represented the most significant change (Figure 1B). Then, we sorted the 56 proteins according to their differential expression with logFC and found that IGFBP7 was the most significant among the proteins related to collagen-containing extracellular matrix (Figure 1C). Pan-cancer analysis based on TCGA data revealed that IGFBP7 was significantly upregulated in GC, esophageal cancer, and pleomorphic glioblastoma, among other cancer tissues, while its expression was downregulated in bladder cancer, renal cell carcinoma, and cervical cancer (Figure 1D). Further analysis of paired tumor and normal samples from the TPM-formatted TCGA-STAD dataset revealed that IGFBP7 mRNA expression levels were also elevated in GC (Figure 1E).

By performing a logrank test on the prognostic outcomes between the high and low IGFBP7 mRNA expression groups, it was found that the high-expression group had a lower overall survival (OS) (HR = 1.690, 95%CI:1.145–2.493) (Figure 1F), progression-free interval (PFI) (HR = 1.600, 95%CI:1.103–2.320), and disease-specific survival (DSS) (HR = 1.677, 95%CI:1.081–2.602) (Supplementary Figure S1A) than the low-expression group. The expression of IGFBP7 mRNA was significantly higher in stages II, III, and IV of GC than in stage I and a similar result was observed in the depth of tumor invasion in GC TNM staging (Figure 1G). In addition, it was found that infection with *Helicobacter pylori* was also associated with high expression levels of IGFBP7 mRNA (Supplementary Figure S1B). During immune infiltration analysis, we observed a strong correlation between IGFBP7 and stromal score. We also found a significant correlation between IGFBP7 and immune score (Supplementary Figure S1C). Furthermore, we discovered a pronounced correlation between IGFBP7 and immune cells such as NK cells (Supplementary Figure S1D).

IHC staining was then performed on tissue microarrays from 80 paired GC and adjacent normal tissue samples to evaluate the protein expression levels of IGFBP7 (Figure 1H). The results confirmed that IGFBP7 protein levels were significantly higher in GC tissues than in adjacent normal tissues. Moreover, IGFBP7 expression was observed in both the infiltrative and expanding subtypes of GC (Figure 1I) but was particularly elevated in the infiltrative type, suggesting that IGFBP7 may be associated with the malignancy of GC.

At the protein level, GC was divided into a low group (0–6) (n = 44, 55%) and a high group (7–12) (n = 36, 45%) based on the staining range and intensity of IGFBP7. By analyzing the relationship between the scores and clinical parameters of GC (Table 1), it was found that the IGFBP7 protein level was related to sex, with lower expression in males than in females (*p* = 0.003). It was also significantly associated with TNM staging, with a clear correlation between the severity of infiltration depth (*p* = 0.026), lymph node metastasis (*p* = 0.030), distant metastasis (*p* = 0.008), and the score. In addition, it was significantly related to vascular cancer embolus (*p* = 0.027). The results suggest that high expression of IGFBP7 protein and mRNA is significantly associated with tumor progression and poor survival rate in GC. 

### 3.2. Single-Cell Analysis Revealed the Expression Characteristics of IGFBP7 in Gastric Cancer

**Figure 2:**
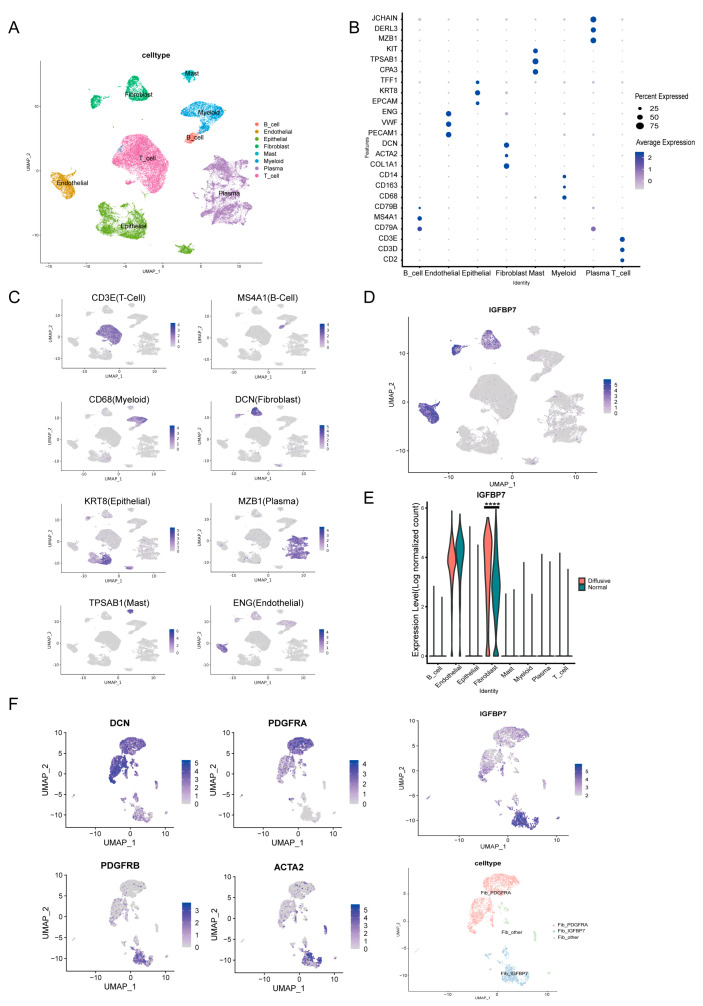
Single−cell analysis revealed the expression characteristics of IGFBP7 in gastric cancer. (**A**) UMAP clustering plot of cells, with different colors representing different cell types. (**B**) Bubble plot showing the expression of marker genes in different cell subtypes. (**C**) UMAP plot showing the expression of individual marker genes. (**D**) UMAP plot showing the expression of IGFBP7. (**E**) Violin plots showing the expression of IGFBP7 in different cell types. (**F**) UMAP plot showing the expression of marker genes and the expression of PDGFRA and IGFBP7 in fibroblast subtypes, in which ACTA2 is a marker for myofibroblasts. ****: *p* < 0.0001.

To further investigate the distribution of IGFBP7 in GC tissues at the single-cell resolution, a scRNA-seq dataset (GSE183904) was obtained from GEO. We selected 6 cases of diffusive GC and 10 normal tissue samples from single-cell data for our study. After quality control and batch effect correction, a total of 45,878 cells were classified into 8 clusters (Figure 2A). Cell types were annotated with typical marker genes, including epithelial cells (EPCAM, TFF1, and KRT8), T cells (CD2, CD3E, and CD3D), plasma cells (JCHAIN, DERL3, and MZB1), B cells (CD79A, CD79B, and MS4A1), fibroblasts (ACTA2, COL1A1, DCN, and PDGFRB), myeloid cells (CD14, CD163, and CD68), endothelial cells (VWF, ENG, and PECAM1), and mast cells (KIT, CPA3, and TPSB2) (Supplementary Table S1). The bubble plot shows the expression of each marker gene in each cell cluster (Figure 2B). The gene mapping plot shows that the marker genes are enriched in the target cell clusters (Figure 2C). It was observed that IGFBP7 was mainly expressed in endothelial cells and fibroblasts, while its expression was relatively low in other cell types (Figure 2D). Notably, fibroblasts in tumor tissue exhibited significantly higher expression levels of IGFBP7 compared to those in normal tissue (*p* < 0.0001) (Figure 2E). 

CAF is mainly divided into extracellular matrix remodeling, myofibroblast, and immune regulatory subgroups based on the expression of specific marker genes [16]. CAFs with high levels of α-SMA expression are often defined as myofibroblastic CAFs (myCAFs) [17]. Based on the abundance of α-SMA, PDGFRB, and PDGFRA, CAFs can be further divided into two subgroups. The positive expression of α-SMA (α-SMA^+^) represents the myCAFs, with higher expression of PDGFRB in the α-SMA^+^ subgroup, while PDGFRA shows more expression in the SMA-subgroup. The IGFBP7^+^ CAFs were predominantly localized to the α-SMA and PDGFRB region on the UMAP projection of CAFs (Figure 2F). It was found that myCAFs express more IGFBP7 than inflammatory CAFs (iCAFs) [18], indicating that IGFBP7^+^ CAFs belong to the subset of myCAFs. 

### 3.3. IGFBP7 Tends to Exert Its Function in the Extracellular Matrix 

**Figure 3:**
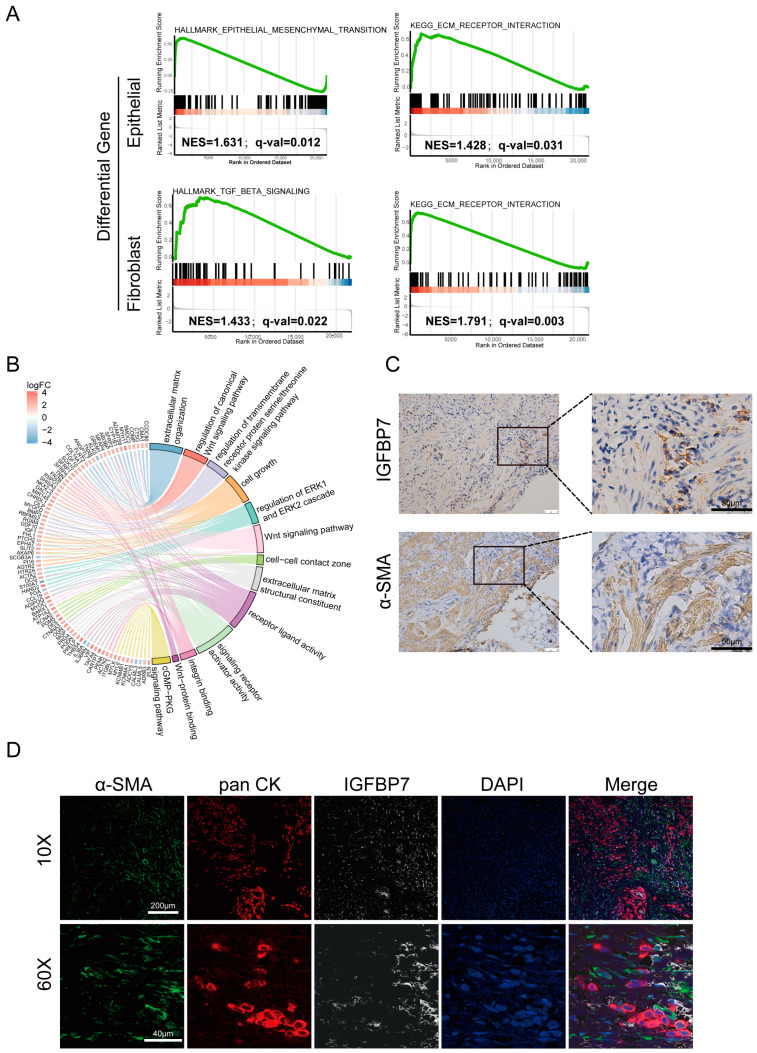
IGFBP7 tends to exert its function in the extracellular matrix. (**A**) GSEA of HALLMARK and KEGG pathways for differentially expressed genes in cancer and adjacent non-cancerous fibroblast subtypes, as well as epithelial cell subtypes. (**B**) Differential gene analysis based on logFC was performed on the low-expression group and high-expression group of IGFBP7 in TCGA, and joint GO and KEGG analysis was conducted on the identified differential gene set. (**C**) Consecutive sections of infiltrative GC tissue showing IGFBP7 (top) and α-SMA (bottom) expression in the same area. (**D**) Multiplex immunofluorescence of infiltrative GC tissue sections: green for α-SMA, red for pan-CK, white for IGFBP7, and blue for DAPI.

From the scRNA-seq data described above, differentially expressed genes (DEGs) were screened between tumor and normal tissues in both fibroblasts and epithelial cells. The upregulated DEGs were subjected to gene set enrichment analysis (GSEA) using the Hallmark gene sets [19]. The results show that genes involved in TGF-β signaling were significantly altered in fibroblast DEGs, while genes involved in EMT signaling were significantly altered in epithelial cell DEGs (Figure 3A). Similarly, KEGG pathway analysis of the DEGs showed that extracellular matrix receptor interaction was significantly enriched in both fibroblast and epithelial cells. Based on the above analysis, we speculate that IGFBP7 is a secreted matrix protein that may serve as a mediator for interaction between GC cells and CAFs. In the TCGA-STAD dataset, based on the expression levels of IGFBP7, samples were divided into two groups: a low-expression group and a high-expression group. Differential gene analysis was performed on the two groups, and the DEGs with |logFC| greater than 2 were subjected to joint GO and KEGG analysis based on logFC (Figure 3B). Similar to the results of GSEA, extracellular matrix composition and ligand activation were enriched, and in addition, regulation of the Wnt pathway and cell growth were enriched.

Consecutive sections of the same GC tissue were used to analyze the correlation between α-SMA and IGFBP7 expression (Figure 3C). IGFBP7 was deposited in varying degrees near the area of α-SMA^+^ fibroblasts. It was also deposited near the extracellular matrix and tumor cells. Then, multiplex immunofluorescence (mIF) was performed in sections of GC tissue; α-SMA was used to label CAFs, and pan-CK was used to label tumor cells. At low magnification (10×), it could be observed that IGFBP7 protein was mainly expressed in the α-SMA^+^ CAFs, which are present in the stromal area, whereas there was less detectable expression of IGFBP7 in panCK^+^ tumor cells. At high magnification (60×), it could be seen that IGFBP7 was deposited in the area of CAFs and partially deposited around tumor cells, indicating that it may act on tumor cells (Figure 3D). However, FAP^+^ CAFs do not express IGFBP7 (Supplementary Figure S1E).

### 3.4. GC Cell-Derived TGF-β1-Activated NFs and Upregulated IGFBP7 Expression via the P-Smad2/3 Pathway

**Figure 4:**
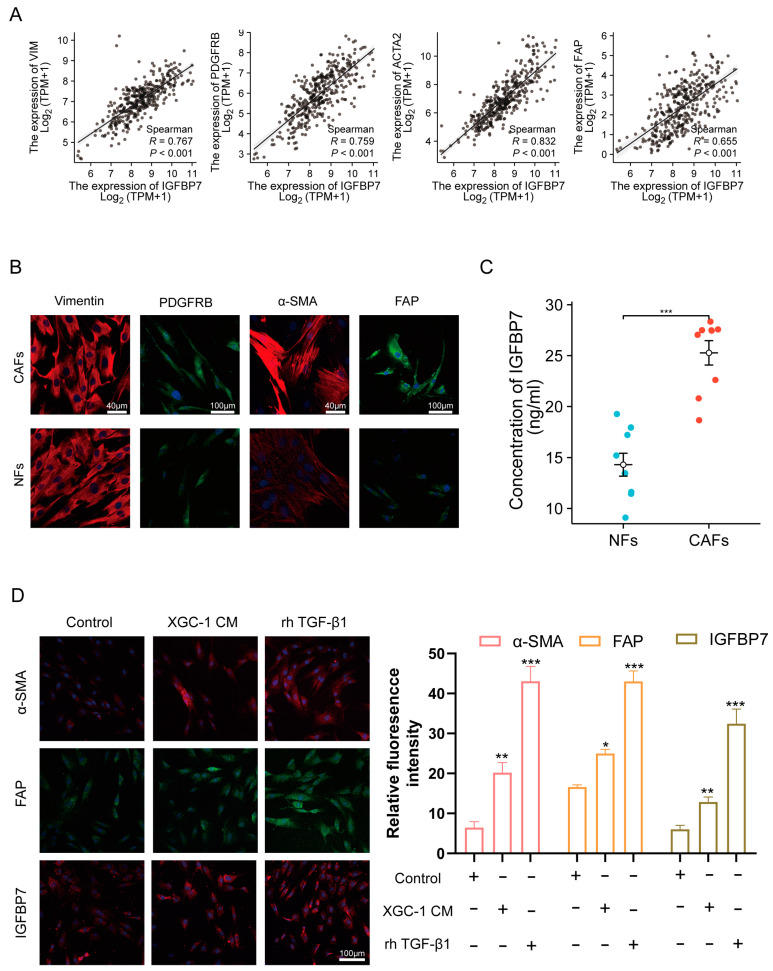
IGFBP7 is associated with activated CAF. (**A**) Correlation analysis of IGFBP7 expression with VIM, PDGFRB, ACTA2, and FAP expression in TCGA-STAD dataset. (**B**) Immunofluorescence (IF) staining of VIM, PDGFRB, ACTA2, and FAP in primary cancer-associated fibroblasts (CAFs) and normal fibroblasts (NFs). (**C**) ELISA analysis of IGFBP7 expression in the culture supernatants of primary CAFs and NFs. (**D**) XGC-1 induces NFs to transform into CAFs through secretion of TGF-β1, *: *p* < 0.05, **: *p* < 0.01, and ***: *p* < 0.001.

The TCGA-STAD database was analyzed to explore the correlation between IGFBP7 and CAF marker genes, including α-SMA, PDGFRB, FAP, and stromal marker vimentin (VIM). IGFBP7 expression was significantly positively correlated with the expression of CAF marker genes. A significant correlation was observed between IGFBP7 and α-SMA (R = 0.821, *p* < 0.001), as well as PDGFRB (R = 0.767, *p* < 0.001) (Figure 4A). In order to validate at the cellular level, primary fibroblasts were extracted from GC surgical specimens. Based on the expression of vimentin, it can be seen that the extracted primary purity was relatively high, and the staining intensity of CAFs’ α-SMA, PDGFRB, and FAP was significantly stronger than that of NFs’ (Figure 4B). The culture supernatants of fibroblasts with the same density were extracted after 24 h of culture, and the protein level of IGFBP7 was analyzed using ELISA (Figure 4C). It is clear that CAFs have a higher expression level, indicating that the results of the scRNA-seq data analysis are correct.

NFs were treated with 30% conditioned medium (XGC-1 CM) from the GC cell line XGC-1, 20ng/mL of recombinant human TGF-β1 (rh TGF-β1), or a control group treated with the same proportion of PBS medium for 48 h. NFs treated with XGC-1 CM and rh TGF-β1 expressed significantly increased protein levels of the CAF marker genes α-SMA and FAP, as well as IGFBP7 (Figure 4D). Meanwhile, we examined the mRNA levels of IL11, IL6, CXCL14, and LIF, which are markers of iCAF [20]. We found that XGC CM and rh TGF-β1 increased the expression levels of IL11 and LIF while inhibiting the expression of IL6. However, they had no effect on the expression of CXCL14 (Supplementary Figure S2).

**Figure 5:**
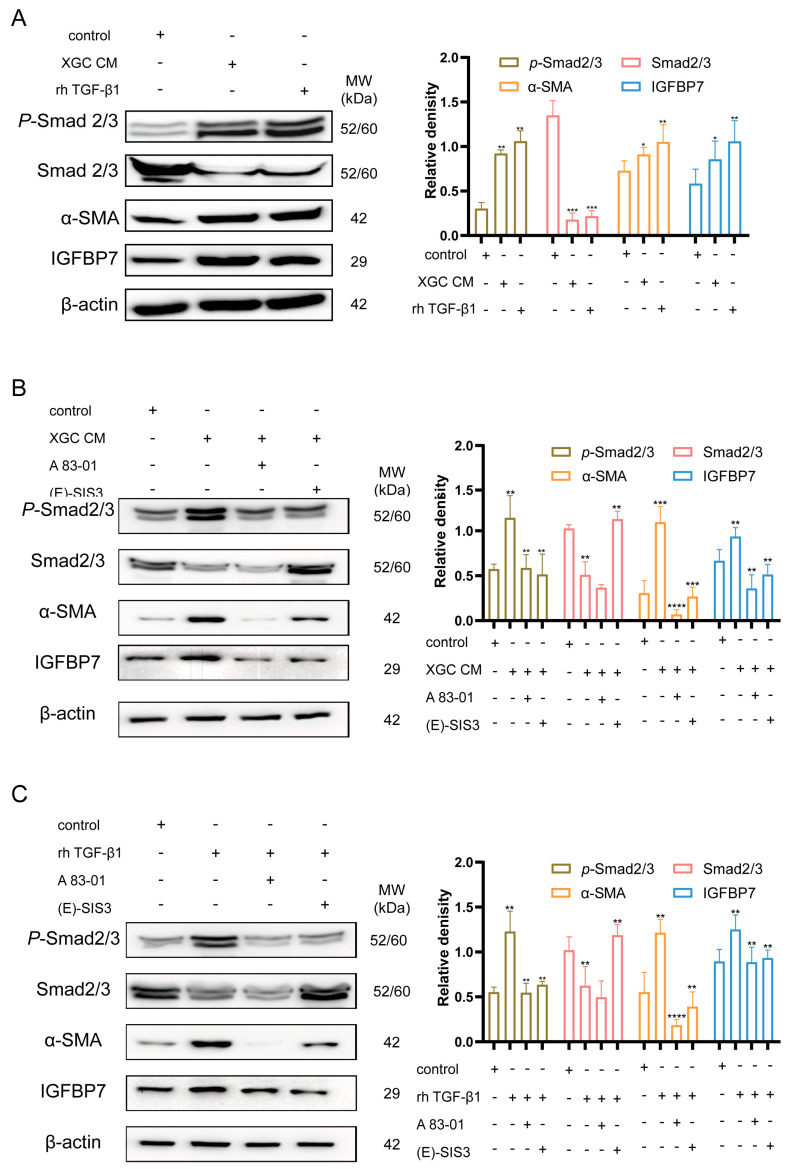
TGF-β1 induces fibroblast expression of IGFBP7. (**A**) XGC-1 promotes fibroblasts to secrete IGFBP7 through the secretion of TGF-β1. (Human recombinant IGFBP7 (rh-IGFBP7), XGC conditioned medium (XGC CM). (**B**,**C**) TGF-β1 receptor inhibitor (A 83-01) and Smad3 phosphorylation inhibitor ((E)SIS3) significantly suppressed the upregulated expression levels of α-SMA and IGFBP7 induced by XGC-1 CM and rh TGF-β1. *: *p* < 0.05, **: *p* < 0.01, ***: *p* < 0.001, and ****: *p* < 0.0001. Original western blots have been presented in File S1.

After treatment with the experimental group, the downstream genes of TGF-β1, i.e., total Smad2/3 protein, were downregulated, while phosphorylated Smad2/3 (*P*-Smad2/3) was upregulated. Similarly, α-SMA and IGFBP7 were upregulated due to the activation of the TGF-β1/Smad2/3 pathway (Figure 5A). Furthermore, treatment with TGF-β1 receptor inhibitor (A 83-01) and Smad3 phosphorylation inhibitor ((E)SIS3) resulted in significant suppression of the increased expression levels of α-SMA and IGFBP7 induced by rh TGF-β1 and XGC-1 CM (Figure 5B,C).

### 3.5. CAF-Derived IGFBP7 Enhances the Metastatic and Stemness Ability of GC

IGFBP7 is primarily induced by the TGF-β1 pathway and secreted into the matrix by CAFs, potentially affecting tumor cells. We investigated the effect of IGFBP7 on GC cells. Two cell lines, XGC-1 and MGC-803, were divided into four groups: (1) Negative control (NC) group, in which the cells were treated with PBS of the same concentration; (2) Recombinant human IGFBP7 (rh-IGFBP7) group, in which the cells were continuously treated with culture medium containing 200 ng/mL of rh-IGFBP7 protein; (3) CAF CM group, in which the cells were treated with 30% CM made from CAFs; and (4) CAF-IGFBP7 CM group, in which the cells were treated with 30% CM made from CAFs overexpressing IGFBP7 (Supplementary Figure S1F). All groups were continuously cultured for 72 h before being subjected to further experiments.

**Figure 6:**
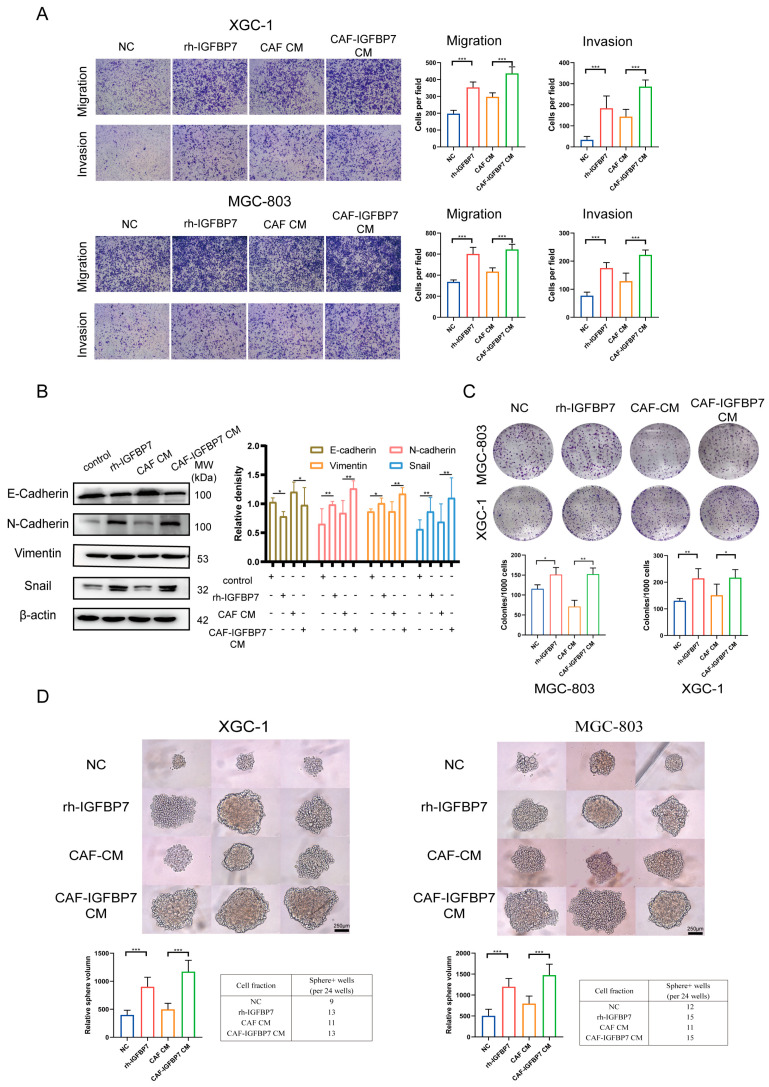
CAF-derived IGFBP7 enhances the metastatic and stemness ability of GC. (**A**) IGFBP7 promotes GC migration and invasion ability. (**B**) IGFBP7 induces EMT in GC cells. (**C**) IGFBP7 enhances the clonogenicity of GC cells. (**D**) IGFBP7 promotes GC sphere-forming ability. CAF IGFBP7 overexpression CM (CAF-IGFBP7 CM). *: *p* < 0.05, **: *p* < 0.01, and ***: *p* < 0.001. Original western blots have been presented in File S1.

The XGC-1 cells treated with rh-IGFBP7 had significantly enhanced migration and invasion abilities compared to the NC group. The CAF-IGFBP7 CM group also exhibited significantly increased migration and invasion abilities compared to the CAF CM group. Similar results were observed in the repeated experiments on MGC-803 (Figure 6A). The XGC-1 was continuously treated with rh-IGFBP7 and CAF-IGFBP7 CM for 96 h. The expression of E-cadherin was downregulated, while the expression of N-cadherin, vimentin, and Snail, the mesenchymal cell markers, was upregulated by exogenous and CAF-derived IGFBP7 protein in XGC-1 (Figure 6B). 

Furthermore, we investigated the effect of IGFBP7 on tumor growth. The results of the colony formation assay showed that the number of colonies was significantly increased in the rh-IGFBP7 group compared with the NC group and in the CAF-IGFBP7 CM group compared with the CAF-CM group. However, there was no significant difference in colony number between the NC group and the CAF CM group or between the rh-IGFBP7 and CAF-IGFBP7 CM groups (Figure 6C). Similar to the colony formation assay, the volume and number of cell spheres were significantly increased in the rh-IGFBP7 group compared with the NC group and in the CAF-IGFBP7 CM group compared with the CAF-CM group. However, while the number of cell spheres was similar between the CAF-CM group and the NC group and between the CAF-IGFBP7 CM group and the rh-IGFBP7 group, the size of cell spheres was significantly increased (Figure 6D). 

**Figure 7:**
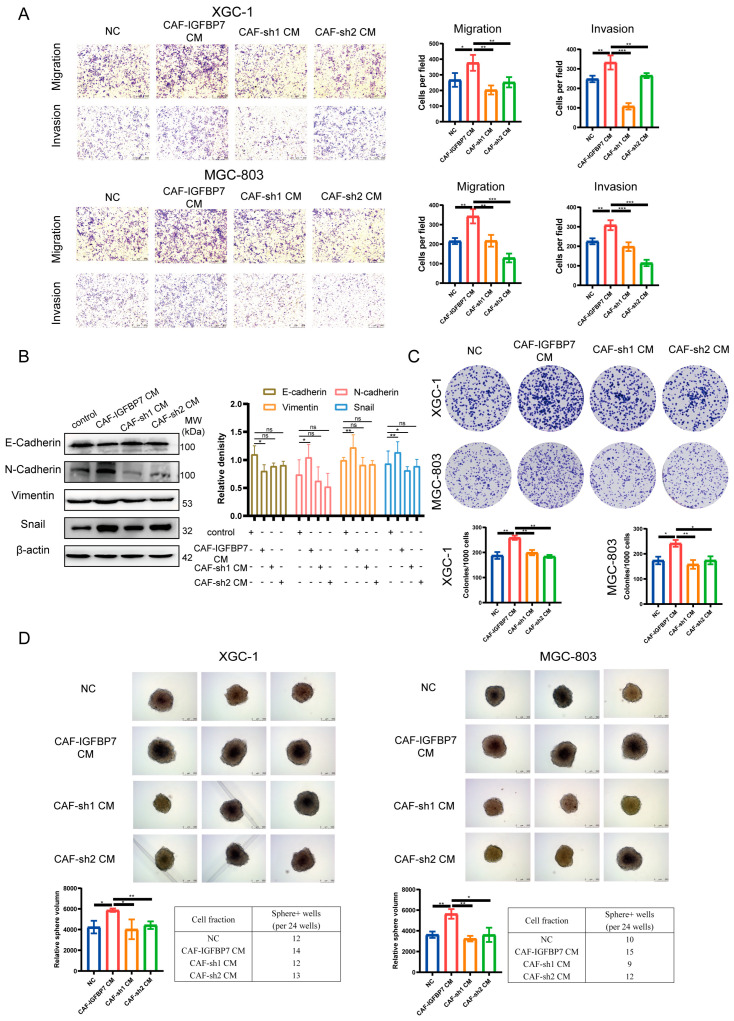
Silencing IGFBP7 in CAFs resulted in the loss of migratory, invasive, and stemness ability of GC induced by CAF-IGFBP7 CM. The conditioned media collected from CAFs with IGFBP7 knockdown (CAF-sh1 CM and CAF-sh2 CM) reversed the migration and invasion (**A**) induced by CAF-IGFBP7 CM, as well as the EMT changes (**B**), clonogenicity (**C**), and sphere-forming ability (**D**) of GC cells. *: *p* < 0.05, **: *p* < 0.01, ***: *p* < 0.001, and ns: not significant. Original western blots have been presented in File S1.

Subsequently, we knocked down IGFBP7 in CAFs (Supplementary Figure S1G) and collected their conditioned media to treat XGC-1 and MGC-803 (CAF-sh1 CM and CAF-sh2 CM). The results showed that compared to CAF-IGFBP7 CM, the enhanced migratory and invasive abilities of GC cells were significantly inhibited (Figure 7A). Similarly, there were no significant changes in the expression of EMT-related markers (E-cadherin, N-cadherin, vimentin, Snail) when comparing the CAF CM with CAF-sh1 CM and CAF-sh2 CM. In comparing CAF-IGFBP7 CM with CAF-sh1 CM and CAF-sh2 CM, N-cadherin, vimentin, and Snail were significantly upregulated, while E-cadherin showed no significant change (Figure 7B). Furthermore, the reduction in clonogenicity and the weakened sphere-forming ability of tumor cells were induced by sh1 CM and sh2 CM compared to CAF-IGFBP7 CM (Figure 7C,D). These results provide evidence that IGFBP7 significantly enhances the migratory and stemness abilities of GC.

## 4. Discussion

The complexity of TME is one of the important challenges faced in the treatment of GC. TME is a complex system composed of various cells and molecules, such as tumor cells, endothelial cells, immune cells, fibroblasts, and matrix, that have complex interactions and regulatory relationships [21]. This leads to the diversity of GC treatment strategies but also makes the design and implementation of treatment more difficult. Increasing evidence emphasizes the indispensable contribution of TME to disease progression and treatment resistance [22]. CAFs promote the progression of GC through reshaping ECM, promoting angiogenesis, and other aspects [23]. In GC, the excessive accumulation of CAFs is related to the increase in tumor size, infiltration, and metastasis ability [24].

IGFBP7 is a secreted protein whose abnormal expression has been found in many types of cancer and which has different clinical values in non-cancerous diseases. Interestingly, the role of IGFBP7 in cancer progression is still controversial. Research findings demonstrate that IGFBP7 exhibits dual effects, i.e., both anti-cancer and pro-cancer in different types of tumors. In liver cancer, IGFBP7 induces anti-tumor effects by binding to the IGF1 receptor (IGF1R) to inhibit IGF signaling [25]; in colon cancer, IGFBP7 down-regulates EMT to promote liver metastasis [26]. Conversely, IGFBP7 is considered a poor prognostic factor in GC, and the increased expression of IGFBP7 is associated with immune infiltration in GC [27,28]. The expression of IGFBP7 is an independent prognostic indicator of decreased survival in esophageal and colorectal cancer patients [29,30]. In addition, in esophageal adenocarcinoma, patients with DNA methylation in the IGFBP7 promoter region often exhibit better survival rates [31]. The diverse functions exhibited by IGFBP7 in different types of cancer can indeed be confusing. However, based on our findings, we speculate that the observed differences between colorectal cancer and gastric cancer may be attributed to the complex tumor microenvironment and the intricate interactions between various cell types. Additionally, it is possible that the effects of IGFBP7 are context-dependent and may vary according to the stage and type of cancer.

CAFs are highly heterogeneous and cannot be simply classified as one type. Two major types of CAFs have been identified in pancreatic cancer: iCAFs and myCAFs [17]. According to this classification, we defined α-SMA^+^CAF as myCAF. Through data analysis, we discovered that IGFBP7^+^ CAF mostly falls into the myCAF category. It is worth noting that IGFBP7 is also associated with immune infiltration, suggesting a potential link between IGFBP7^+^ CAF and immune responses. The exact nature of this relationship is currently unclear. In our study, we found a correlation between IGFBP7 and NK cells. It has been reported that increased infiltration of NK cells in the tumor microenvironment is associated with improved response to immune checkpoint blockade [32]. However, in recent gastric cancer research, studies have demonstrated that targeting both PDGFRA and PDGFRB in the tumor stroma can reverse the immunosuppressive microenvironment, leading to improved effectiveness of immune checkpoint inhibitors (ICIs) [33]. Moreover, the expression of α-SMA in CAF seems to be correlated with the response to ICIs in gastric cancer [34]. Based on our speculation, we propose that α-SMA^+^, PDGFRB^+^, and IGFBP7^+^ CAFs play a role in tumor immunotherapy response. 

IGFBP7 expressed by endothelial and fibroblast cells may have multiple interaction mechanisms in GC. The main function of endothelial cells is to form the blood vessel wall, promote angiogenesis, and maintain vascular stability [35], thereby providing support for tumor growth and metastasis [36]. CAFs are connective tissue cells that mainly secrete matrix molecules such as hyaluronan to maintain tissue structure and function [37]. In the GC microenvironment, CAFs act directly and indirectly on GC cells by participating in fibrotic reactions and producing collagen fibers and matrix molecules [38]. Understanding the mechanisms of action of endothelial and CAFs can provide new ideas and strategies for the prevention and treatment of GC.

GSEA analysis showed that the TGF-β pathway was activated, as well as intercellular matrix protein receptor interactions. We hypothesize that IGFBP7 may be related to the activation of the TGF-β pathway; TGF-β is often secreted by tumor cells in the tumor microenvironment to reshape the tumor microenvironment and immune response [39]. The TGF-β pathway consists of both classical and non-classical pathways, with the classical pathway promoting the expression of type I collagen and α-SMA, as well as the deposition of ECM through Smad2/3 phosphorylation [40]. TGF-β is often secreted by cancer cells, thereby regulating the tumor microenvironment [41]. NFs are incubated with XGC-1 CM and rhTGF-β1 to induce their transformation into CAFs. The classical pathway activated by TGF-β1 leads to an increase in Smad2/3 phosphorylation and upregulation of α-SMA and IGFBP7 expression. We observed that XGC-1 CM and rhTGF-β1 have an impact on the expression of iCAF markers, which could be attributed to the activation of other signaling pathways by TGF-β1 [42,43].

The IGFBP7 protein was mainly located in the stromal region, where it is attached to and acts around the tumor cells, consistent with our single-cell data analysis results. TCGA correlation expression and continuous section staining of α-SMA and IGFBP7 showed that their expression patterns were basically consistent, further confirming IGFBP7 expression in the activated fibroblast region. Studies have shown that the IGFBP7–CD93 axis interacts with endothelial cells, which can alter the normal morphology of tumor microenvironment blood vessels, leading to drug resistance. Blocking IGFBP7 can normalize blood vessels and drug delivery [44]. This suggests that IGFBP7 affects tumor progression in multiple ways.

Furthermore, endogenous and exogenous IGFBP7 could promote the migration and invasion ability of GC. IGFBP7 could induce EMT transition in XGC-1, downregulating E-cadherin expression and upregulating N-cadherin, vimentin, and Snail expression. This has been reported to induce EMT via IGFBP7 in human alveolar epithelial cells and renal tubular epithelial cells [45,46], but this study represents the first time it has been discovered in GC. Similarly, IGFBP7 could enhance the tumor cells’ ability to grow on plates and form spheres, indicating a positive effect on the cloning and growth ability of GC cells.

CAFs can regulate the growth, infiltration, and invasion of GC cells by secreting a series of factors. Among them, IGFBP7, an important molecule, plays an important role in tumor development. It can promote cell proliferation and apoptosis, thus, having a tumor-promoting effect. Specifically, IGFBP7 secreted by CAFs can bind to receptors on the surface of tumor cells, leading to the activation of downstream oncogenic signaling pathways. In addition, we speculate that IGFBP7 can bind to extracellular matrix molecules such as collagen, thereby altering the cell–matrix interaction and further promoting infiltration and metastasis. Therefore, IGFBP7 secreted by CAFs plays an important role in the development of GC, and understanding its regulatory mechanism can help to deepen our understanding of the interactions and regulatory mechanisms in the GC microenvironment.

## 5. Conclusions

**Figure 8:**
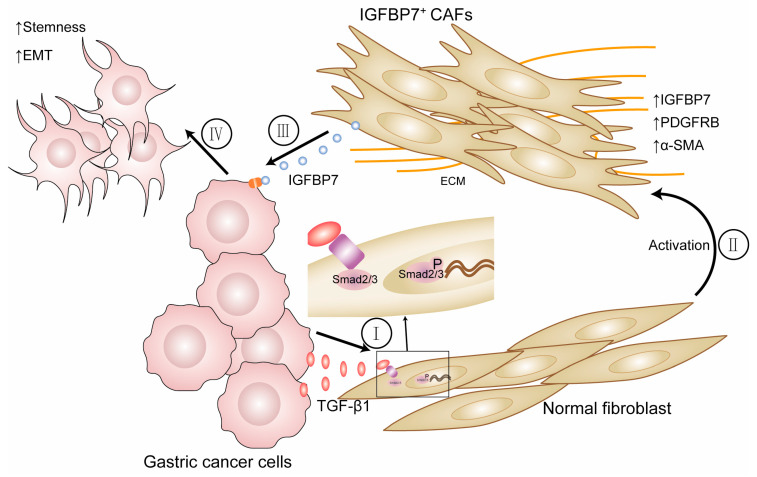
(I, II) GC cells activate NFs by secreting TGF-β1, which activates and transforms them into CAFs. Then, TGF-β1 increases the expression of IGFBP7 by activating the P-Smad2/3 pathway. (III, IV) IGFBP7 enters the tumor microenvironment to promote the metastatic and stemness ability of GC cells.

In summary, we investigated the interaction between CAFs and GC cells using bioinformatics analysis and a series of experiments. The conclusion drawn was that IGFBP7 is expressed at higher levels in infiltrating and poorly differentiated GC, and based on clinical data from TCGA and tissue microarrays, higher expression of IGFBP7 is associated with more advanced clinical staging. Secondly, based on GC tissue scRNA-seq data and IHC, it was suggested that IGFBP7^+^ CAFs are associated with the aggressive biological behavior of poorly differentiated GC. Finally, through cellular-level experiments, it was found that this interaction involves GC cells secreting TGF-β1, which acts on NFs, causing them to transform into the myofibroblast subtype of CAFs. These CAFs then express IGFBP7 and interact with GC cells, potentially playing an important role in the development of GC (Figure 8). The specific mechanism by which IGFBP7 induces biological changes in gastric cancer is still subject to further research. 

## Figures and Tables

**Table 1:** The baseline clinical data of the immunohistochemical score of IGFBP7.

Group	IGFBP7 IHC Score		*p* Value
	Low (0–6)	High (7–12)	
Numbers, n (%)	44 (55%)	36 (45%)	
Gender			0.003 **
male	40 (50%)	23 (28.7%)	
female	4 (5%)	13 (16.2%)	
Age, mean ± sd	64.636 ± 10.625	61.389 ± 12.133	0.206
Depth of invasion			0.026 *
T1 and T2	8 (10%)	0 (0%)	
T3	14 (17.5%)	13 (16.2%)	
T4	22 (27.5%)	23 (28.7%)	
Lymph node			0.030 *
N0	13 (16.2%)	4 (5%)	
N1	8 (10%)	6 (7.5%)	
N2	14 (17.5%)	8 (10%)	
N3	9 (11.2%)	18 (22.5%)	
Metastasis			0.008 **
M0	44 (55%)	29 (36.2%)	
M1	0 (0%)	7 (8.8%)	
Clinical stage			0.390
II	15 (18.8%)	11 (13.8%)	
II–III	11 (13.8%)	14 (17.5%)	
III	18 (22.5%)	11 (13.8%)	
Lymphovascular invasion			0.027 *
yes	20 (31.7%)	9 (14.3%)	
no	14 (22.2%)	20 (31.7%)	
Nerve invasion			0.687
yes	14 (23.7%)	9 (15.3%)	
No	20 (33.9%)	16 (27.1%)	

(* *p* < 0.05, ** *p* < 0.01).

## Data Availability

Data supporting present findings are available from the corresponding author upon reasonable request.

## Supplementary Materials

The following supporting information can be downloaded at: https://www.mdpi.com/article/10.3390/cancers15153965/s1. Supplementary Figure S1: Correlation between IGFBP7 expression and clinical information and other protein expression; Supplementary Figure S2: RT-PCR was conducted to examine the expression levels of IL11, CXCL14, IL6, and LIF of normal fibroblasts after treating with XGC-1 CM and rhTGF-β1. Table S1: he canonical marker genes used for annotating major cell types; File S1: Original western blots. References [47,48,49,50,51,52] are cited in Supplementary Materials.
