# Supplementary material for: Crosstalk between Cancer Cells and Cancer-Associated Fibroblasts Mediated by TGF-β1–IGFBP7 Signaling Promotes the Progression of Infiltrative Gastric Cancer
Source: Cancers (Basel). 2023 Aug 4;15(15):3965. doi: 10.3390/cancers15153965 (PMC10417438; doi:10.3390/cancers15153965)
Supplement: Supplementary file 1 [file cancers-15-03965-s001.zip › cancers-2407916-supplementary/Supplementary Figures.pdf]

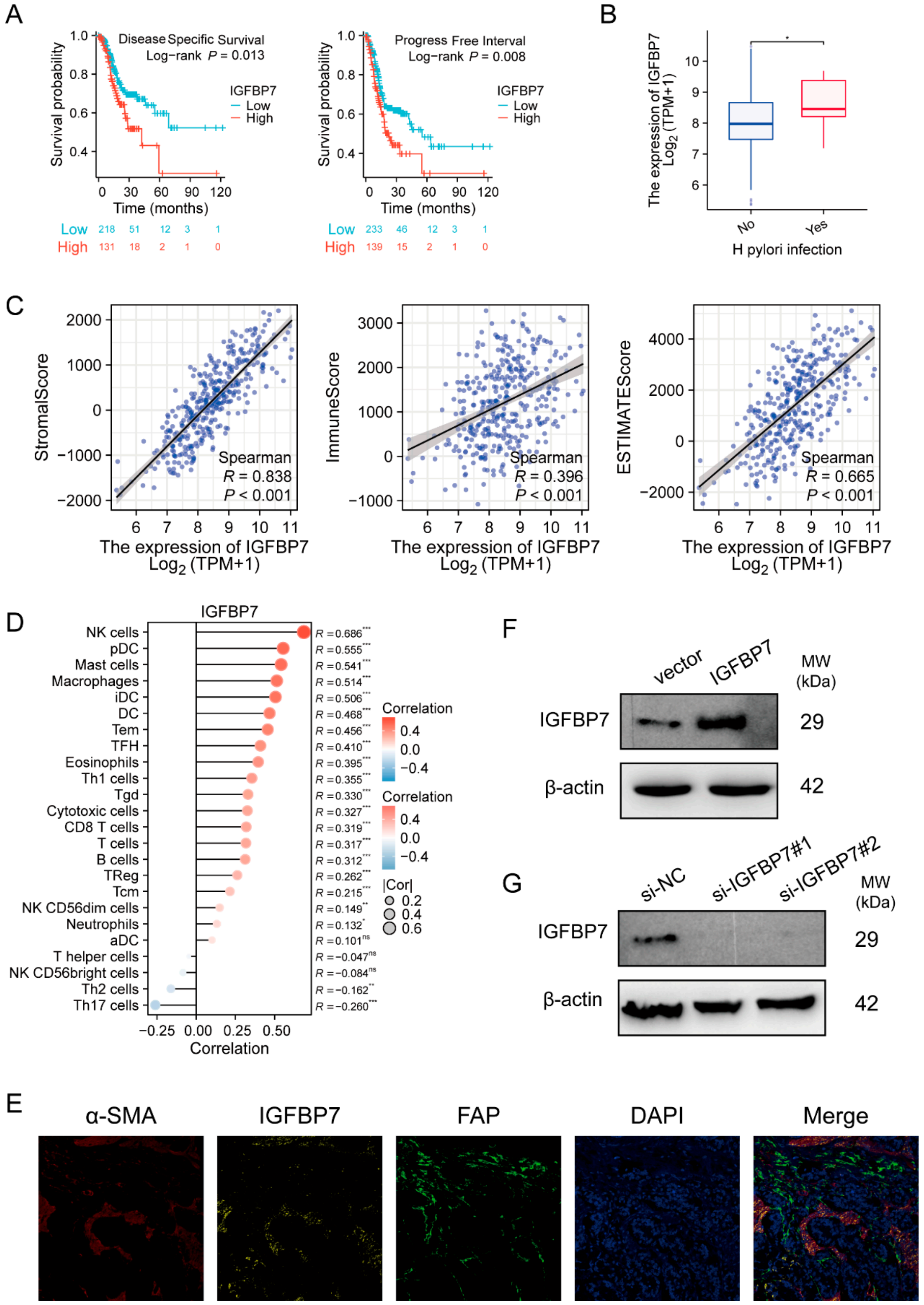

Supplementary Figure S1. (A) Higher levels of IGBP7 mRNA are negatively associated with Disease Specific Survival and Progression Free Interval, and (B) positively associated with *Helicobacter pylori* infection. (C) The ESTIMATE algorithm revealed a positive correlation between the expression level of IGFBP7 and stromal cells, immune cells, and tumor purity scores. (D) IGFBP7 expression was

analyzed using ssGSEA in relation to immune cell infiltration.(E) Multiple immunofluorescence analysis was performed to investigate the expression relationship between α-SMA (red), IGFBP7 (yellow), and FAP (green).(F,G) Western blot assess the efficiency of IGFBP7 overexpression and knockdown.

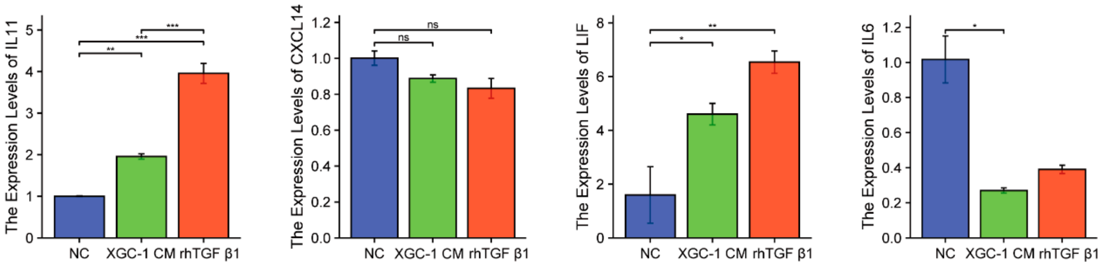

Supplementary Figure S2. RT-PCR was conducted to examine the expression levels of IL11, CXCL14, IL6, and LIF of normal fibroblasts after treating with XGC-1 CM and rhTGF-β1.
