# Supplementary material for: Crosstalk between Cancer Cells and Cancer-Associated Fibroblasts Mediated by TGF-β1–IGFBP7 Signaling Promotes the Progression of Infiltrative Gastric Cancer
Source: Cancers (Basel). 2023 Aug 4;15(15):3965. doi: 10.3390/cancers15153965 (PMC10417438; doi:10.3390/cancers15153965)
Supplement: Supplementary file 1 [file cancers-15-03965-s001.zip › cancers-2407916-supplementary/Supplementary Table S1.pdf]

Supplementary Table S1.

The canonical marker genes used for annotating major cell types.

B cells	T cells	Epithelial cells	Plasma cells	Fibroblasts	Myeloid cells	Endothelial cells	Mast cells
CD79A[1]	CD2[1]	EPCAM[2]	JCHAIN[3]	ACTA2[1]	CD14[2]	VWF[1]	KIT[4]
CD79B[4]	CD3E[1]	TFF1[5]	DERL3[6]	COL1A1[2]	CD163[2]	ENG[1]	CPA3[1]
MS4A1[1]	CD3D[1]	KRT8[2]	MZB1[6]	DCN[3]	CD68[2]	PECAM1[3]	TPSB2[4]
				PDGFRB[4]			

1. Zhang, M.; Hu, S.; Min, M.; Ni, Y.; Lu, Z.; Sun, X.; Wu, J.; Liu, B.; Ying, X.; Liu, Y. Dissecting transcriptional heterogeneity in primary gastric adenocarcinoma by single cell RNA sequencing. *Gut* **2021**, *70*, 464-475, doi:10.1136/gutjnl-2019-320368.
2. Wang, R.; Dang, M.; Harada, K.; Han, G.; Wang, F.; Pool Pizzi, M.; Zhao, M.; Tatlonghari, G.; Zhang, S.; Hao, D.; et al. Single-cell dissection of intratumoral heterogeneity and lineage diversity in metastatic gastric adenocarcinoma. *Nat Med* **2021**, *27*, 141-151, doi:10.1038/s41591-020-1125-8.
3. Luo, H.; Xia, X.; Huang, L.B.; An, H.; Cao, M.; Kim, G.D.; Chen, H.N.; Zhang, W.H.; Shu, Y.; Kong, X.; et al. Pan-cancer single-cell analysis reveals the heterogeneity and plasticity of cancer-associated fibroblasts in the tumor microenvironment. *Nat Commun* **2022**, *13*, 6619, doi:10.1038/s41467-022-34395-2.
4. Zhao, W.; Jia, Y.; Sun, G.; Yang, H.; Liu, L.; Qu, X.; Ding, J.; Yu, H.; Xu, B.; Zhao, S.; et al. Single-cell analysis of gastric signet ring cell carcinoma reveals cytological and immune microenvironment features. *Nat Commun* **2023**, *14*, 2985, doi:10.1038/s41467-023-38426-4.
5. Kumar, V.; Ramnarayanan, K.; Sundar, R.; Padmanabhan, N.; Srivastava, S.; Koiwa, M.; Yasuda, T.; Koh, V.; Huang, K.K.; Tay, S.T.; et al. Single-Cell Atlas of Lineage States, Tumor Microenvironment, and Subtype-Specific Expression Programs in Gastric Cancer. *Cancer Discov* **2022**, *12*, 670-691, doi:10.1158/2159-8290.CD-21-0683.
6. Patil, N.S.; Nabet, B.Y.; Muller, S.; Koeppen, H.; Zou, W.; Giltnane, J.; Au-Yeung, A.; Srivats, S.; Cheng, J.H.; Takahashi, C.; et al. Intratumoral plasma cells predict outcomes to PD-L1 blockade in non-small cell lung cancer. *Cancer Cell* **2022**, *40*, 289-300 e284, doi:10.1016/j.ccell.2022.02.002.
