# Supplementary figures and images for: Crosstalk between Cancer Cells and Cancer-Associated Fibroblasts Mediated by TGF-β1–IGFBP7 Signaling Promotes the Progression of Infiltrative Gastric Cancer
Source: Cancers (Basel). 2023 Aug 4;15(15):3965. doi: 10.3390/cancers15153965 (PMC10417438; doi:10.3390/cancers15153965)

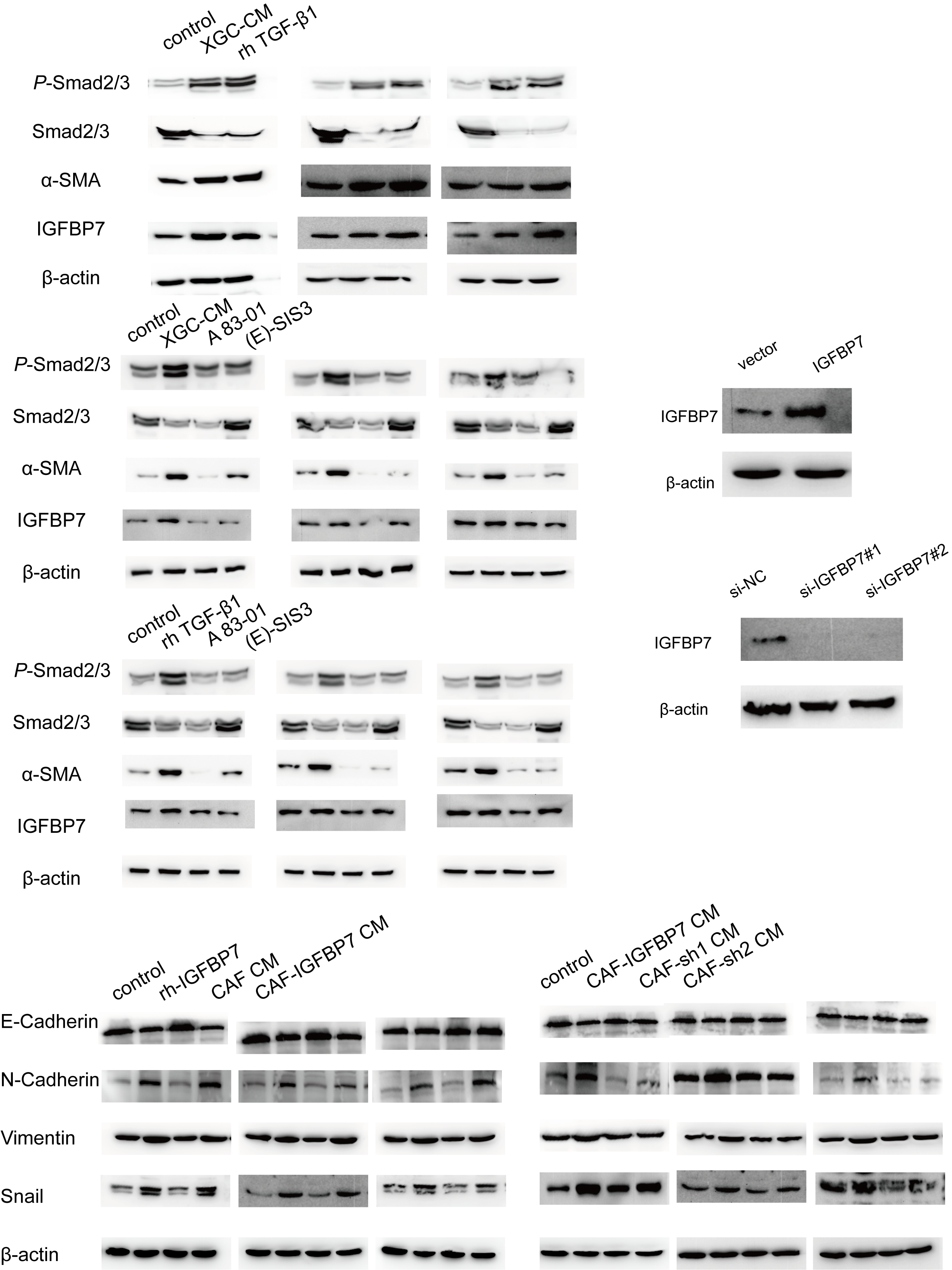

Supplement: Supplementary file 1 [file cancers-15-03965-s001.zip › cancers-2407916-supplementary/File S1. Original western blots.tif]
